# Assessing Visitor Expectations of AI Nursing Robots in Hospital Settings: Cross-Sectional Study Using the Kano Model

**DOI:** 10.2196/59442

**Published:** 2024-11-27

**Authors:** Aimei Kang, XiuLi Wu

**Affiliations:** 1 Department of Nursing, Wuhan Asia Heart Hospital Affiliated to Wuhan University of Science and Technology, Wuhan, China; 2 Institute of Nursing Research, School of Medicine, Wuhan University of Science and Technology, Wuhan, China

**Keywords:** nursing robot, artificial intelligence, Kano model, demand survey, nursing, care robots, nursing management

## Abstract

**Background:**

Globally, the rates at which the aging population and the prevalence of chronic diseases are increasing are substantial. With declining birth rates and a growing percentage of older individuals, the demand for nursing staff is steadily rising. However, the shortage of nursing personnel has been a long-standing issue. In recent years, numerous researchers have advocated for the implementation of nursing robots as a substitute for traditional human labor.

**Objective:**

This study analyzes hospital visitors’ attitudes and priorities regarding the functional areas of artificial intelligence (AI) nursing robots based on the Kano model. Building on this analysis, recommendations are provided for the functional optimization of AI nursing robots, aiming to facilitate their adoption in the nursing field.

**Methods:**

Using a random sampling method, 457 hospital visitors were surveyed between December 2023 and March 2024 to compare the differences in demand for AI nursing robot functionalities among the visitors.

**Results:**

A comparative analysis of the Kano attribute quadrant diagrams showed that visitors seeking hospitalization prioritized functional aspects that enhance medical activities. In contrast, visitors attending outpatient examinations focused more on functional points that assist in medical treatment. Additionally, visitors whose purpose was companionship and care emphasized functional aspects that offer psychological and life support to patients.

**Conclusions:**

AI nursing robots serve various functional areas and cater to diverse audience groups. In the future, it is essential to thoroughly consider users’ functional needs and implement targeted functional developments to maximize the effectiveness of AI nursing robots.

## Introduction

With the gradual increase in the number of older and chronically ill individuals worldwide, the shortage of human nursing resources has emerged as a significant global issue [1]. This shortage not only compromises the quality of care services but also poses challenges to the efficiency and sustainability of the overall medical system [2,3]. Integrating artificial intelligence (AI) and mobile medicine into clinical practice is becoming more prevalent to address the current inadequate amount of nursing personnel. For instance, Wu et al [4] demonstrated that AI-based personalized interventions can enhance the self-management of patients with type 2 diabetes in primary care settings, thereby improving behavioral effectiveness. Additionally, Turkish scholars have used AI to develop predictive models that assist physicians in the prediagnosis and differential diagnosis of children’s mental illnesses in clinical practices [5]. Among the various intelligent technologies, AI nursing robots have garnered significant attention from the nursing community. These robots are intelligent devices that combine AI algorithms with robotics technology to deliver nursing services and support [6]. Over the past decade, AI nursing robots have rapidly advanced within the medical field, showing substantial potential to enhance nursing services and alleviate the burden on nursing staff [7]. In nursing practices, robots have taken over specific labor-intensive tasks traditionally performed by human nurses, proving them to be practical auxiliary tools in various medical scenarios [8]. For example, during the COVID-19 pandemic, scholars such as Yang et al [9] suggested that AI nursing robots can serve as a tool to reduce the risk of virus transmission and mitigate the impact on frontline health care practitioners. In addition to the benefits that a socially assistive robot can provide to individuals with cognitive impairment [10], there exists a multifunctional robot known as Pepper. This versatile robot can be applied in various sectors, including industry, entertainment, and patient care, offering innovative solutions and enhancing experiences across diverse fields. Its primary functions involve engaging individuals with specific diseases in therapeutic activities, assisting them in completing tasks that enhance their physical functions, and offering emotional support to patients [11].

People’s attitudes and opinions toward new products and technologies are influenced by specific contexts, such as families and schools. Research groups can be diverse, including nurses, nursing managers, and medical students. For instance, a recent survey indicated that caregivers prefer robots to perform simple, highly repetitive tasks [12]. Conversely, medical students hold differing perspectives, believing that nursing robots are incapable of providing patient companionship and that they can only function as assistants to medical staff [13]. Notably, while most researchers agree that patients and their families are the primary beneficiaries of AI nursing robots, there is a scarcity of studies addressing their functional needs regarding these robots. Consequently, this study is grounded in the Kano theoretical model and analyzes hospital visits, offering insights for the development of medical care robots that are safe, efficient, and aligned with clinical requirements.

The Kano model, proposed by Japanese scholar Noriaki Kano in the 1980s, serves to classify user needs and expectations, enabling designers to prioritize the development of product features and functions more effectively [14]. Currently, demand surveys based on the Kano model are widely used in the medical field, proving to be an effective tool for assessing both patient and worker satisfaction [15,16]. Specifically, the Kano model categorizes functional attitude attributes into six distinct types: attractive attributes, one-dimensional attributes, must-be attributes, indifferent attributes, reverse attributes, and questionable results [15]. Table 1 summarizes the characteristics of each attribute. When applying the Kano model for product analysis, the general priority ranking of functional requirements is as follows: must-be attributes>one-dimensional attributes>attractive attributes>indifferent attributes [17]. In product development, it is advisable to prioritize functions that possess must-be, one-dimensional, or attractive attributes. Indifferent attributes may be appropriately discarded after carefully considering factors such as market demand, user feedback, resource constraints, and the competitive environment [18].

**Table 1.** Characteristic table of Kano attributes.

Attribute name	Attribute characteristics
Attractive attribute	If the function exceeds the user’s expectations, the function is highly perfect, and user satisfaction and acceptance will increase significantly. Without the function, user satisfaction and acceptance will not decrease significantly.
One-dimensional attribute	The presence of a particular function enhances satisfaction and acceptance, while its absence leads to a decrease in satisfaction and acceptance.
Must-be attribute	The presence of a specific function does not directly enhance satisfaction and acceptance, but its absence results in a decline in satisfaction and acceptance.
Indifferent attribute	The presence or absence of a specific function does not impact satisfaction and acceptance.
Reverse attribute	Without a particular function, satisfaction would be higher.
Questionable result	The user does not understand a question or answer it correctly.

## Methods

### Study Design

**Figure 1.**
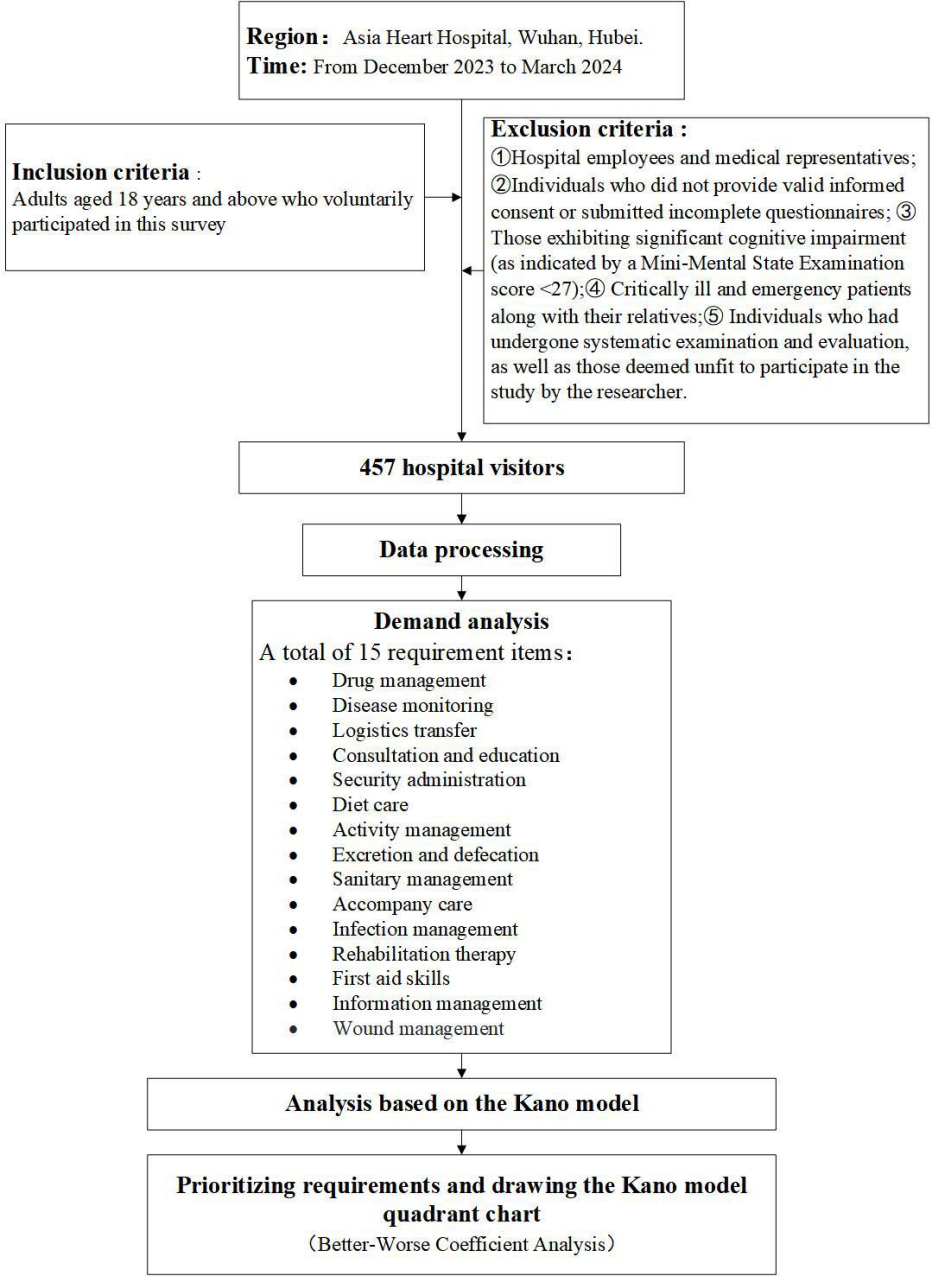
Research flowchart.

This study employed a cross-sectional survey design to assess the needs of the respondents. Subsequently, the data was analyzed using the Kano model to evaluate demand attributes and importance coefficients. Figure 1 displays the research flowchart.

### Participants

A random sampling method was employed to select visitors from the Wuhan Asia Heart Hospital as the primary subjects of this study, irrespective of their region or age. The minimum required number of questionnaires was determined using formula 1.


(1) $$N=\frac{Z^{2}\sigma^{2}}{d^{2}}$$

In this formula, σ represents the SD of the population, typically set at 0.5, while *d* refers to the CI of 50%. Given that the Kano model is employed to assess users’ opinions regarding a particular function, which is inherently subjective, we opted for a wider CI of 90%. Consequently, the confidence level statistic *Z* was determined to be 1.64, and the calculated sample size was a minimum of 68 cases. To mitigate the risk of bias in the study, after thorough discussions among the researchers and considering the hospital’s foot traffic, it was decided to increase the number of questionnaires distributed to 500. This adjustment aimed to balance the limitations of sample size, time, and resources, thereby facilitating the attainment of reliable research results.

The inclusion criteria for study participants were adults aged 18 years and older who voluntarily participated in this survey. The exclusion criteria included hospital employees and medical representatives, individuals who did not provide valid informed consent or whose questionnaires were incomplete, those with significant cognitive impairment (indicated by a Mini-Mental State Examination score<27), critically ill and emergency patients along with their relatives, individuals who had undergone systematic examination and evaluation, and those deemed by the researcher to be unfit for participation in the study.

### Survey Tools

The survey tool consisted of a questionnaire we designed, primarily divided into two sections: a general information survey and a Kano survey scale. The general information questionnaire aimed to gather essential demographic data, including sex, marital status, age, education level, prior exposure to AI nursing robots, acceptance of AI nursing robots for nursing services, and the reasons for visiting the hospital. The second section features the Kano survey scale, which assessed the desired functional requirements of AI nursing robots. Through an integrated analysis of the potential for robots to perform nursing tasks in clinical settings, we delineated 15 nursing fields using card induction classification (see Textbox 1). Consequently, the Kano survey scale was constructed, comprising the 15 items. Each item consisted of two questions—one framed positively and the other negatively—to evaluate hospitalized patients’ attitudes and preferences regarding the functionalities of AI nursing robots. Each question encompassed five dimensions: very much liked, taken for granted, indifferent, reluctantly accepted, and very disliked. The attributes of each factor were then classified based on the survey results.

Textbox 1. **Classification of nursing fields and a summary of the functional points.**

**1. Drug management**

- Intravenous dispensing and transport
- Drug distribution

**2. Disease monitoring**

- Determination of vital signs
- Warning of critical condition
- Access monitoring

**3. Logistics transfer**

- Patient transfer and goods consignment

**4. Consultation and education**

- Outpatient guidance, consultation, and registration
- Explanation of nursing measures
- Guidance on disease knowledge and health behavior promotion

**5. Security administration**

- Prevention of falls, bed falls, and other accidental injuries
- Danger warning and disaster prevention

**6. Diet care**

- Dining guidance and supervision
- Nutritional status monitoring and diet planning
- Nursing measures related to gastric tube maintenance

**7. Activity management**

- Positioning and assisted ambulation
- Activity training

**8. Excretion and defecation**

- Handling of excreta, cleansing, and documentation
- Toilet assistance

**9. Sanitary management**

- Personal hygiene and cleaning
- Bed linen and clothing maintenance and replacement

**10. Accompany care**

- Interpersonal interaction
- Social skills training

**11. Infection management**

- Ward cleaning, disinfection, and sterilization
- Biological sampling
- Microbial identification and antimicrobial efficacy evaluation

**12. Rehabilitation therapy**

- The restoration of physical function and exercise
- Perceived training
- Daily functional training and treatment

**13. First aid skills**

- Cardiopulmonary resuscitation
- Oxygen absorption and sputum absorption

**14. Information management**

- Collection and storage of hospital admission personnel information
- Information retrieval

**15. Wound management**

- Replacing wound dressings, alleviating pain, and protecting the wound

We employed a Cronbach α coefficient to assess the reliability of the questionnaire and conducted a factor analysis to evaluate its validity. Given that the Kano questionnaire includes both forward and reverse questions for each item, the reliability and validity analyses were performed separately for these two types of questions. The results indicated that the Cronbach α coefficient for the forward questions was 0.952, while the Cronbach α for the reverse questions was 0.958, demonstrating high reliability for the scale. The Kaiser-Meyer-Olkin values for the forward and reverse questions were 0.945 and 0.954, respectively. Additionally, the results of Bartlett’s test of sphericity revealed a significance level of *P*＜.001, suggesting that the validity and reliability of the questionnaire are both acceptable.

### Data Collection and Analysis

From December 2023 to March 2024, we conducted random sampling at the outpatient department of the Wuhan Asia Heart Hospital in Hubei, China, selecting a total of 510 hospital visitors, of whom 482 agreed to participate in our study. After identifying the survey subjects, we employed face-to-face surveys to collect data, with each questionnaire taking between 10 to 30 minutes to complete. Subsequently, two nursing graduate students used Excel 2019 (Microsoft Corporation) to verify and correct the data. Upon obtaining the original dataset, we calculated the Better value and Worse value for each functional area using IBM SPSS 26.0 (IBM Corp). Finally, we employed Prism 10 (GraphPad Software) to create the Kano demand quadrant diagram, using the Better value as the ordinate and the absolute value of Worse as the abscissa, thereby incorporating each function point for a more intuitive classification display. In the Kano demand quadrant chart, the attributes that fall into the first quadrant are one-dimensional attributes, those in the second quadrant are attractive attributes, those in the third quadrant are indifferent attributes, and those in the fourth quadrant are must-be attributes.

### Ethical Considerations

Approval for this study was granted by the Ethics Committee at Wuhan University of Science and Technology (No. 2024‐096). The research was carried out in alignment with the guidelines established in the Declaration of Helsinki and its later revisions, along with other relevant ethical standards. Before participating, all individuals gave informed consent, the questionnaires were filled out anonymously, and we complied with the principles of voluntary withdrawal and the avoidance of harm. Participants in this study did not receive any form of compensation for their involvement.

## Results

### Basic Information Statistics Table of Survey Objects

The hospitals surveyed were primarily located in Wuhan, Hubei. A total of 482 questionnaires were distributed, of which 24 were deemed disqualified and subsequently removed, resulting in a final collection of 457 questionnaires. The effective recovery rate for the questionnaires was 94.8% (457/482). Male participants constituted 48.4% (221/457) of the survey, while female participants represented 51.6% (236/457). Among the participants, 10 were younger than 18 years old, 37 were between 18 and 25 years old, 85 were aged between 26 and 30 years, 60 participants were between 31 and 40 years old, and 100 participants were between 41 and 50 years old. Additionally, 154 participants were aged between 51 and 60 years, and the remaining 21 participants were older than 60 years old. Further details are provided in Table 2.

**Table 2.** Basic information statistics table.

Categories	Participants (n=457), n (%)	
Sex		
Male	221 (48.4)	
Female	236 (51.6)	
Marital status		
Married	201 (44)	
Unmarried	256 (56)	
Age (years)		
18-20	10 (2.2)	
20-25	27 (5.9)	
26‐30	85 (18.6)	
31‐40	60 (13.1)	
41‐50	100 (21.9)	
51‐60	154 (33.7)	
>60	21 (4.6)	
Educational background		
Primary school and below	30 (6.6)	
Junior middle school	120 (26.3)	
High school/technical secondary school	184 (40.3)	
Undergraduate/junior college	92 (20.1)	
Master’s degree or above	31 (6.8)	
Have you used intelligent nursing robots before?		
Yes	229 (50.1)	
No	228 (49.9)	
Whether to accept the AI^a^ nursing robot to provide nursing services		
Yes	313 (68.5)	
No	103 (22.5)	
Uncertainty	41 (9)	
Reason for hospital visit		
Hospitalization	154 (33.7)	
Outpatient examination	159 (34.8)	
Accompany care	144 (31.5)	

a AI: artificial intelligence.

### Requirements Result Analysis

#### Classification of Functional Demand Attributes of AI Nursing Robots

**Figure 2.**
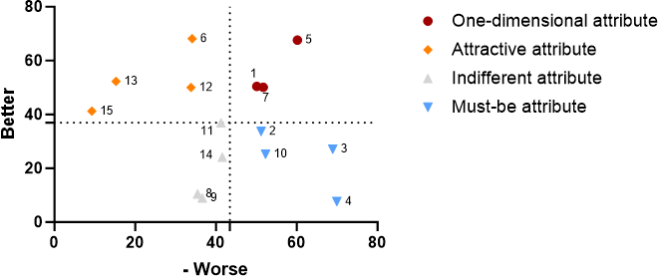
Overall personnel demand quadrant chart for artificial intelligence nursing robot functions.

According to the overall personnel Kano demand quadrant diagram, a total of four items were classified within the must-be attribute quadrant, three items fell into the one-dimensional attribute quadrant, four items were categorized in the attractive attribute quadrant, and the remaining four items were placed in the indifferent attribute quadrant (Figure 2). This indicates that hospital visitors in this survey prioritized the functional needs of AI nursing robots in the following order: disease monitoring, accompany care, logistics transfer, and consultation and education>drug management, activity management, and security administration>dietcare, rehabilitation therapy, first aid skills, and wound management>infection management, information management, excretion and defection, and sanitary management. This result suggests that if AI nursing robots can effectively provide functions such as condition monitoring, companion care, logistics transfer, and consultation and education, along with support in areas like medication management, safety management, activity management, rehabilitation physiotherapy, nutritional care, and first aid skills, user satisfaction and recognition will likely increase. In contrast, hospital visitors appear to be less concerned about functions such as infection management and excretion care, possibly due to the personal privacy implications associated with these tasks. Therefore, when designing and implementing nursing robots in the future, it is crucial to focus on how to protect user privacy.

#### Hospital Visitors‘ Functional Demand Preferences for AI Nursing Robots Based on Reason for Attending the Hospital

**Figure 3.**
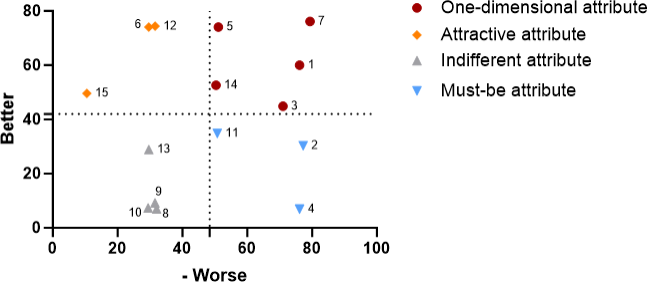
Demand quadrant chart for artificial intelligence nursing robot functions among visitors seeking hospitalization.

**Figure 4.**
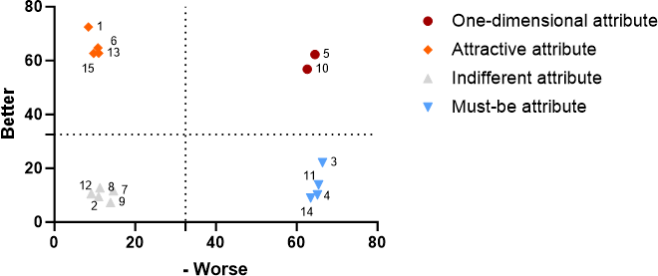
Demand quadrant chart for artificial intelligence nursing robot functions among visitors seeking outpatient examination.

**Figure 5.**
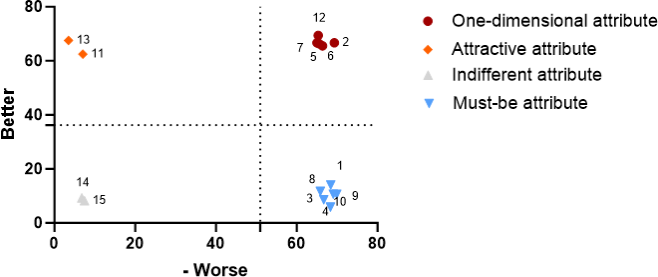
Demand quadrant chart for artificial intelligence nursing robot functions among visitors for accompany care.

In the general information questionnaire, the survey participants were categorized into three groups based on their primary reason for visiting the hospital. Among these, 154 individuals sought hospitalization, 159 individuals attended outpatient examinations, and 144 individuals came for companionship and care. To determine whether the purpose of the hospital visit influenced the wanted functional requirements of nursing robots, we constructed Kano quadrant diagrams illustrating the functional requirements of AI nursing robots for hospital visitors for three distinct purposes, as depicted in Figures 3-5. Figure 3 indicated that the priority of functional requirements for AI nursing robots aimed at hospitalized patients was as follows: infection management, consultation and education, and disease monitoring>security administration, information management, activity management, drug management, and logistics transfer>wound care, diet care, and rehabilitation physiotherapy. The least prioritized functions included first aid skills, life care, companionship care, and excretion care. For visitors seeking outpatient examinations, the prioritized functions of AI nursing robots were logistics transfer, infection management, information management, and consultation and education, followed by security administration and accompanying care, and then drug management, diet care, first aid skills, and wound care. Rehabilitation physiotherapy, excretion care, activity management, sanitary management, and condition monitoring were of lesser priority (Figure 4). Conversely, visitors whose purpose was to provide companionship and care prioritized these functions for AI nursing robots in the following order: drug management, excretion and defecation, logistics transfer, sanitary management, companion care, and consultation and education>activity management, rehabilitation physiotherapy, disease monitoring, diet care, and safety education>first aid skills and infection management>information management and wound management (Figure 5).

## Discussion

### Summary

This article thoroughly examines hospital visitors’ current use of AI nursing robots and their preferred specific functional requirements. Given the rapid advancements in AI and mobile medical technologies, it is essential to gain a comprehensive understanding of the preferences and needs of hospital visitors regarding these robots. Such insights will provide a solid foundation for future policy development and the implementation of advanced technologies, thereby facilitating the progressive enhancement of intelligent nursing practices in China. Through this research, we aim to assist nurses in fully leveraging the potential of AI in clinical settings, promoting the seamless integration and efficient transformation of technology within clinical applications.

### Positive Acceptance Attitude

Overall, respondents had a positive attitude toward the clinical application of AI nursing robots. In this study, 50.1% (229/457) of hospital visitors reported having used AI nursing robots, while 68.5% (313/457) expressed a strong interest in and willingness to explore the clinical applications of these robots. This indicates that hospital visitors possess a certain level of awareness regarding the potential value and advantages of AI nursing robots and are open to the integration of this emerging technology into clinical care, which aligns with the perspectives of nursing leaders on robotics [19]. However, some respondents also voiced concerns related to nursing robots, including issues of privacy exposure and operational safety, and this concern is corroborated by the findings of Wong et al [20]. Recently, social robots using AI technology have made advancements in memory training, medication guidance, and emotional communication services, with some of their functionalities becoming integrated into people’s daily lives [21]. However, reports indicate these robots are not yet equipped to deliver such complex services reliably [22]. To mitigate the potential risks that robots may pose to patients and their families, actively promoting the intelligent nursing processes within Chinese medical institutions is essential. Additionally, there is a need for the gradual enhancement of relevant legislation, the standardization of industry standards for the production of service robots, and the strengthening of institutional support and policy guarantees. Ultimately, these measures will foster the overall development of China’s intelligent care industry.

### Different Purposes Affect the Choice of Hospital Visitors’ Preferred Functional Requirements for AI Nursing Robots

By examining the overall demand survey results, we can conclude that participants exhibit distinct differences in their functional attribute requirements for AI nursing robots based on their reasons for visiting the hospital. Individuals seeking hospitalization are generally more receptive to AI nursing robots that enhance their medical care, such as condition monitoring, consultation and education, medication management, safety oversight, and rehabilitation physiotherapy. In contrast, those attending outpatient examinations tend to prioritize AI nursing robots that assist with medical treatment logistics, information management, and companion care, showing less interest in rehabilitation, physiotherapy, activity management, and life care functionalities. Furthermore, individuals seeking companionship care emphasize the companionship functions of AI nursing robots, expressing a desire for these robots to offer psychological support and assistance in daily activities, including necessary help with excretion and other daily living tasks. In light of the findings from this Kano survey, we recommend that designers and developers undertake targeted functional developments tailored to medical institutions’ specific functional scenarios when creating nursing service robots [12]. Furthermore, when medical institutions implement and deploy AI nursing robots, they should carefully consider the specific application scenarios and tasks, ensuring the effective use of various functional areas. For instance, in hospital outpatient departments, service robots equipped with guidance and consultation capabilities should be prioritized. In care wards, those with condition monitoring functions should take precedence. In rehabilitation wards, robots designed for rehabilitation physiotherapy should be prioritized, and particularly in home or community settings, robots with companion care functionalities should be deployed.

Currently, the application of AI nursing robots is primarily focused on areas such as logistics and transportation [23], infection wards [24], and rehabilitation [25], where they have demonstrated promising results. Considering the functional needs expressed by survey respondents regarding AI nursing robots, it is crucial to emphasize the expansion and enhancement of these robots’ functional capabilities in the future. Specifically, there should be a focus on the development of features related to consultation, education, and companion care. This approach will better address the needs of patients in hospitals and ultimately enhance the clinical application value of AI nursing robots.

### Limitations

This study has several limitations. First, the research subjects were confined to the Wuhan Asia Heart Hospital, and the potential influence of factors such as hospital grade, region, education level, salary level, and medical insurance on the experimental results, as well as the ethical issues associated with the use of robots, were not examined. Second, while the purpose for attending the hospital may be more varied and diverse in practice, this study only included three primary purposes. Finally, this study primarily focuses on AI nursing robots; future research should also consider other intelligent products.

### Conclusion

As a product needs analysis tool, the Kano model can be used to identify and classify the impact of various needs on user acceptance and satisfaction [26]. This study examined multiple perspectives from hospital visitors, including inpatients, outpatients, and individuals accompanying patients, and it investigated the differences in the prioritization of hospital visitors’ needs for AI nursing robots based on these differing visitation purposes. We identified that people have a positive attitude toward the use of AI nursing robots and that the preferred functional requirements of AI nursing robots differ depending on their reason for going to the hospital. This provides valuable insights for robot developers and guidance for hospitals and individuals. Notably, the development of new functionalities for nursing robots encompasses multiple disciplines, including health care, engineering, and human-computer interaction. We anticipate future interdisciplinary collaboration to explore practical solutions to the challenges faced by AI-assisted nursing, thereby accelerating the advancement of this field. The transformation of these solutions into practical functions within actual products and apps will foster the development and application of nursing robot technology, driving innovation and progress in the medical sector.

## Abbreviations

AI: artificial intelligence
